# Pharmacists’ Perceptions of Physician–Pharmacist Collaboration—A 2022 Cross-Sectional Survey in Poland

**DOI:** 10.3390/healthcare11172444

**Published:** 2023-08-31

**Authors:** Iwona Wrześniewska-Wal, Jarosław Pinkas, Janusz Ostrowski, Mateusz Jankowski

**Affiliations:** School of Public Health, Centre of Postgraduate Medical Education, 01-826 Warsaw, Poland

**Keywords:** pharmacist–physician collaboration, pharmaceutical care, community pharmacists, interprofessional collaboration, Poland

## Abstract

Patient-centered care requires close collaboration among multiple healthcare professionals, including physician–pharmacist collaboration (especially as a part of pharmaceutical care). This study aimed to assess pharmacists’ perceptions of physician–pharmacist collaboration as well as to identify factors associated with the willingness to provide pharmaceutical care services in Poland. This questionnaire-based survey was carried out in 2022 among community pharmacists from one of the largest franchise chain pharmacy networks in Poland. Completed questionnaires were received from 635 community pharmacists (response rate of 47.9%). Almost all the pharmacists agreed with the statement that there is a need for physician–pharmacist collaboration (98.2%), and 94.8% declared that pharmacists can help physicians in patient care and pharmacotherapy. Most pharmacists (80%) believed that physicians were not aware of the competencies of pharmacists resulting from Polish law. Patient education (89.9%), detection of polypharmacy (88%), and detection of interactions between drugs and dietary supplements (85.7%) were the most common tasks in the field of pharmaceutical care that can be provided by a pharmacist. Females were more likely (*p* < 0.05) to declare the need for physician–pharmacist collaboration. Age and location of the pharmacy were the most important factors (*p* < 0.05) associated with pharmacists’ attitudes toward physician–pharmacist collaboration.

## 1. Introduction

Patient safety is a key element of healthcare, and its achievement requires close collaboration among the patient, healthcare professionals, and the healthcare system as a whole [1]. Many strategies are used to ensure patient safety, such as standardization of procedures, training of medical staff, monitoring and response to medical errors, incident reporting systems, application of hygiene rules, and ensuring the availability of appropriate equipment and medicines [2,3,4,5]. One of the strategies to increase the efficiency, quality, and accessibility of healthcare is interprofessional cooperation [6].

Including a community pharmacist in a multidisciplinary patient care team and integrating state-of-the-art pharmaceutical services with medical and nursing care are among the most important challenges facing the healthcare system in Poland [7,8].

Pharmacotherapy is the basic element of the treatment of many civilization diseases. Drug-related problems such as noncompliance, under- or over-prescription, side effects, or drug interactions have a significant impact on treatment outcomes. A chance to limit the effect of drug-related problems on treatment outcomes is the involvement of pharmacists in patient care [9]. The definition adopted by the Pharmaceutical Care Network (PCNE) states that “Pharmaceutical Care is a pharmacist’s contribution to the care of individuals to optimize the use of drugs and improve health outcomes” [10]. This is a broad definition with three main elements: (1) detection of actual or potential drug problems and patient assessment, (2) solving real drug problems, and (3) prevention of drug problems [10].

The role of the pharmacist in healthcare has changed. Pharmaceutical care should be equally accessible, and the role of the pharmacist is to ensure that medicines are used effectively and safely, leading to the best possible outcome for the patient [9,10,11]. There are different pharmaceutical care models implemented around the world [12,13]. In many countries, such as Belgium, Finland, Italy, Switzerland, and the United Kingdom (UK), pharmacists provide direct care to patients (such as adherence and support for chronic disease management and medication review). Pharmacists also play a greater role in health promotion and disease prevention, especially in rural areas [13]. In many Organisation for Economic Co-operation and Development (OECD) member countries, community pharmacists’ scope of practice has been further expanded in response to COVID-19. In Poland, the powers of pharmacists have also been extended, e.g., in the field of vaccinations [14].

However, the most important change in Poland introduced by the Act of 16 April 2021 on the profession of pharmacist [15] is the possibility to provide services as part of pharmaceutical care. In the Act, pharmaceutical care is defined as “healthcare services constituting a documented process in which a pharmacist cooperates with a physician and a patient and supervises the course of pharmacotherapy” [15]. The planned pharmaceutical care services, which were included in the report of the Ministry of Health of April 2021, are [15]:

- Drug reviews for adults who take five or more drugs at the same time;
- The New Drug service, enabling support for the patient in starting chronic treatment with a new drug;
- Nutritional advice provided by pharmacists;
- preventive programs, blood pressure measurement in a pharmacy, or determination of the BMI index;
- Flu vaccinations;
- Support in setting up an Internet Patient Account [16].

Despite the law regulation on pharmaceutical care, the current implementation of pharmaceutical care in community pharmacies in Poland is very limited. Previously published data showed that pharmacists were aware of the importance of this pharmaceutical care and confirmed the need to provide pharmaceutical care services and the to regulate legal and organizational issues of pharmaceutical care [17]. However, numerous pharmacists also declared concerns about the implementation of pharmaceutical care, mostly related to a lack of dedicated funding or a lack of knowledge on interprofessional collaboration [18,19]. Information on new competencies gained by the pharmacist was distributed using medical media as well as materials from pharmaceutical chambers and medical chambers [15]. However, there was a limited amount of training on interprofessional collaboration dedicated to pharmacists or physicians. During the COVID-19 pandemic, pharmacists were actively involved in pharmacist-administrated COVID-19 vaccination [14]. Moreover, there is a public debate on the role of pharmacists in the healthcare system in Poland and the mixed-skills approach in healthcare. Currently, there is a lack of a physician–pharmacist collaboration model that can be implemented in Poland. Identification of pharmacists’ perceptions of physician–pharmacist collaboration may provide practical information that can be used by policymakers to prepare interprofessional collaboration guidelines in the Polish healthcare system. 

This study aimed to assess the pharmacists’ perception of physician–pharmacist collaboration as well as to identify factors associated with the willingness to provide pharmaceutical care services in Poland.

## 2. Materials and Methods

This questionnaire-based cross-sectional study was carried out in the last quarter of 2022. A self-prepared questionnaire was sent to all pharmacists from one of the largest franchise chain pharmacy networks in Poland [20]. This franchise chain pharmacy network has approximately 550 pharmacies in all administrative regions in Poland [20]. A dedicated link to the study questionnaire was available using Google Forms and sent to 1327 community pharmacists from all over the country affiliated with one of the largest franchise chain pharmacy networks in Poland [20]. The questionnaire was available online and distributed using an internal communication network manager by the franchise chain pharmacy office. Each participant declared informed consent. Pharmacy technicians and support staff were not included in the study. The study protocol was approved by the local ethics committee (Ethics Committee at the Centre of Postgraduate Medical Education in Warsaw, Poland, decision number 128/2022). 

The study questionnaire included 23 questions, prepared based on the literature review on physician–pharmacist collaboration [7,8,17,18,21,22]. Respondents were asked about the need for physician–pharmacist collaboration, pharmaceutical care services that can be provided by a pharmacist, attitudes toward the implementation of formal physician–pharmacist collaboration in Poland, and areas requiring changes to facilitate physician–pharmacist collaboration. Currently, in Poland, there is a lack of physician–pharmacist collaboration funded by the public payer and provided with health insurance. For this reason, in this study questions on pharmacists’ perception of the collaboration between physicians (in general) and pharmacists were addressed. Respondents were asked about different actions that can be taken as a part of physician–pharmacist collaboration (including pharmaceutical care).

A pilot study was conducted among 16 community pharmacists, who filled out the questionnaire twice, 5–7 days apart. However, validity and reliability of the instrument were not tested, and the values of Cronbach’s alpha or kappa coefficient are not presented in this article. After the analysis of the responses, three questions were rewritten to clarify the text, and one question was removed.

Data were analyzed using SPSS software version 28 (IBM, Armon, NY, USA). Frequencies and proportions were used to present the distribution of categorical variables. Cross-tabulations with chi-square tests were used to compare categorical variables. Multivariable regression analyses were carried out to identify factors associated with the willingness to provide pharmaceutical care services and get involved in physician–pharmacist collaboration. Four independent variables were included in the model: gender, age, having a pharmaceutical specialization, and location of the pharmacy. Covariates were based on the own experience of the authors from research among healthcare professionals and literature review [7,8,17,18,21,22]. The strength of association was presented with an odds ratio (OR) and 95% confidence intervals (CI). The statistical significance level was set at *p* < 0.05.

## 3. Results

### 3.1. Respondents’ Characteristics

Responses were received from 635 community pharmacists in Poland (response rate: 47.9%); 80.9% of respondents were female, and the mean age was 41 +/− 10.0 years. Among the respondents, 32% had a pharmaceutical specialization. Most of the respondents worked in pharmacies located in cities below 100,000 residents, 26.3% worked in pharmacies located in cities from 100,000 to 50,000 residents, 20.3% of respondents worked in pharmacies located in cities above 500,000 residents, and 2.5% of respondents worked in pharmacies located in rural areas.

### 3.2. Pharmacists’ Perception of the Physician–Pharmacist Collaboration

Almost all the pharmacists agreed (strongly agree or rather agree) with the statement that there is a need for physician–pharmacist collaboration (98.2%), and 94.8% declared that pharmacists can help physicians in patient care and the selection of optimal pharmacotherapy (Table 1). Most pharmacists (80%) believed that physicians were not aware of the competencies of pharmacists resulting from Polish law. Patients visiting general practitioners were indicated as a group that can benefit the most (69%) from physician–pharmacist collaboration. Development of guidelines and recommendations on physician–pharmacist collaboration (94%), providing public funding for physician–pharmacist collaboration (86.9%), and inclusion of physician–pharmacist collaboration in medical education programs (81.9%) were indicated as priority activities for the improvement of physician–pharmacist collaboration.

In the opinion of pharmacists, patient education on the use of medical equipment (89.9%), detection of polypharmacy (88%), and detection of interactions between drugs prescribed by a physician and dietary supplements self-ordered by the patient (85.7%) were the most common tasks in the field of pharmaceutical care that can be provided by a pharmacist (Table 1). Moreover, 79.2% of pharmacists declared willingness to provide counseling on lifestyle changes in chronic diseases. Among the respondents, 62% declared willingness to provide pharmacotherapy and adherence/adherence monitoring as a part of physician–pharmacist collaboration/pharmaceutical care (Table 1). More than half of respondents indicated pharmaceutical counseling in minor health problems (55.3%) and detection of prescribing cascade and recommendations for limiting the number of drugs (56.1%) as tasks in the field of pharmaceutical care that can be provided by a pharmacist. 

Female respondents more often declared (Table 2) conviction about the need for physician–pharmacist collaboration (98.8% vs. 95.0%; *p* = 0.01) and belief that pharmacists can help the physicians in patient care (95.9% vs. 90.1%; *p* = 0.01).

Female respondents more often declared willingness to provide services aimed at the detection of polypharmacy (89.9% vs. 80.2%; *p* = 0.003), but male respondents more often declared willingness to perform pharmacotherapy and adherence monitoring (70.2% vs. 60.1%; *p* = 0.04). The percentage of pharmacists who declared willingness to provide services aimed at the detection of polypharmacy, detection of interactions between drugs and dietary supplements, as well as detection of prescribing cascade was the highest among the youngest (under 35 years of age) pharmacists (Table 3). Pharmacists with pharmaceutical specialization less often declared willingness to perform pharmacotherapy and adherence monitoring (56.2% vs. 64.8%; *p* = 0.04). Pharmacists working in rural areas less often declared willingness to perform patient education on the use of medical equipment, pharmacotherapy, and compliance monitoring as well as detection of polypharmacy (Table 3). Details are presented in Table 3. 

Female respondents were more likely (*p* < 0.05) to declare the need for physician–pharmacist collaboration (Table 4).

Female respondents were more likely to declare willingness to provide services aimed at detection of polypharmacy (*p* = 0.002). Pharmacists aged below 55 years of age were more likely to declare willingness to provide 6 of 8 analyzed pharmaceutical care services (Table 5). Pharmacists working in pharmacies located in cities compared to those working in rural areas were more likely to declare willingness to provide services aimed at detection of polypharmacy and patient education on the use of medical equipment (*p* < 0.05). In multivariable regression analysis, there was no impact of having a pharmaceutical specialization on the perception of the need for implementation of pharmaceutical care services. Details are presented in Table 5.

## 4. Discussion

This study revealed that community pharmacists declared their readiness to cooperate with physicians and believed that the implementation of physician–pharmacist collaboration in the healthcare system (including services funded under mandatory health insurance in Poland) can improve the quality of patient care. However, most pharmacists believed that physicians did not know the professional competencies of pharmacists resulting from Polish law. Patient education, detection of polypharmacy, and drug reviews were the most common pharmaceutical care services that pharmacists would like to perform. Lack of guidelines on interprofessional collaboration and limited funding were the most common barriers to physician–pharmacist collaboration. There were significant differences in the perception of pharmaceutical care services that can be provided by a pharmacist by age and community pharmacy location. 

Countries with a healthcare workforce shortage such as Poland are constantly working on skill mix in the healthcare workforce and new law regulations that may increase the range of healthcare services provided by non-physicians [23]. To improve the quality of care and increase the effectiveness of pharmacotherapy, public authorities in Poland increased the scope of professional competence of pharmacists [24]. Based on the Act on the Profession of Pharmacists (December 2020), pharmacists gained a new professional competency, including the ability to provide pharmaceutical care services. Physician–pharmacist collaboration, especially related to drug review, is a key part of pharmaceutical care [9,10]. However, the successful implementation of pharmaceutical care requires cooperation between pharmacists and physicians. Zielińska-Tomczak et al. [7,8], Merks et al. [19], and Bojar et al. [22] showed that community pharmacists in Poland declared positive attitudes toward the implementation of pharmaceutical care and physician–pharmacist collaboration. This study also confirmed that over 98% of community pharmacists in Poland believed that there is a need for physician–pharmacist collaboration. Contrary to most of the previous studies, this study was conducted after the implementation of the new law on the profession of pharmacists in Poland. In this study, 95% of participants believed that pharmacists could help physicians in patient care and the selection of optimal pharmacotherapy, which is also in line with the previously published data [7,8,19,22]. Female respondents were more likely to declare that pharmacists can support physicians in patient care. This observation may result from the fact that over 80% of pharmacists in Poland are female [25]. Moreover, this study also showed that 80% of pharmacists believed that physicians did not know the competencies of pharmacists resulting from Polish law. The justification for this situation should be sought in the dysfunction of the current model of the physician–pharmacist relationship. Bradley et al. [24] proposed a three-level model for assessing physician–pharmacist cooperation: the first level is isolation, the second level is communication, and the third level is cooperation. In Poland, the relationship between pharmacists and physicians is described as isolated [21]. The isolation of these two groups is one of the reasons why in our study as many as 14.5% of pharmacists stated that they had no opinion on the knowledge of physicians regarding the competence of pharmacists under the law. In Poland, there is a generally accepted healthcare culture with a dominant role and responsibility of the physician. Therefore, contacts between physicians and pharmacists are often limited to formal activities (contact to verify the content of the prescription) [26]. Moreover, there is a significant gap in physician–pharmacist communication, which is mostly impersonal and based on phone calls or handwritten notes. Such a style of cooperation was described as anachronistic in Chen T.F.’s research a quarter of a century ago [27]. In Poland, there is also a lack of education on physician–pharmacist collaboration in medical schools. According to the World Health Organization (WHO), interprofessional education is a necessary step in preparing “cooperative practitioners” of health professionals who are better equipped to respond to local needs [28]. 

In this study, most of the pharmacists believed that the greatest benefits (69%) from physician–pharmacist collaboration can be gained by patients who visit a general practitioner. This is confirmed by previously published studies from other countries, which show that the inclusion of pharmacists in primary care probably reduces the number of visits to general practitioners and is associated with fewer visits to emergency departments [29]. 

There are different models of physician–pharmacist collaboration around the world [11,27,30]. In Australia, Canada, and the United Kingdom, pharmacists are valuable members of cross-professional patient care teams because they are medicine experts and can share their knowledge with colleagues to improve medication use and patient safety [30]. However, building a model of collaboration between physicians and pharmacists is a long-term process, and the key elements are trust, interdependence, perceptions of another healthcare professional, skills, interest in collaborative practice, role definition, and communication [30]. In Eastern Europe, the most frequently mentioned barriers to physician–pharmacist collaboration are the lack of knowledge about the services provided in pharmacies, the financing model and the heavy burden on pharmacies, and the lack of private consultation rooms for patients [31]. Findings from this study also confirmed that the development of guidelines on physician–pharmacist collaboration is a key activity to increase the role of pharmacists in healthcare.

The concept of pharmaceutical care promotes physician–pharmacist collaboration based on lasting interprofessional relations [17,19,21]. Out of eight different pharmaceutical care services analyzed in this study, patient education, detection of polypharmacy, and detection of interactions between drugs were the most common services that pharmacists would like to provide. 

Most respondents pointed to the important role of pharmacists in the care of chronically ill patients, including the prevention of medication errors [32]. The World Health Organization (WHO) has defined the concept of Therapeutic Patient Education (TPE) as a process aimed at helping the patient to acquire or maintain the competencies needed to self-manage a chronic disease [33]. In chronic disease, the patient first needs advice on lifestyle changes. In this study, 79.2% of the pharmacists declared their willingness to provide counseling on lifestyle changes. Pharmacists can play an important role in patient education on lifestyle and may provide some lifestyle-related interventions, e.g., smoking cessation [34]. The EuroPharm Forum, in collaboration with the WHO Unit for Tobacco and Health, developed a smoking cessation program introduction document, addressed mainly to national pharmaceutical associations, and smoking cessation guidelines at the pharmacy level [34]. 

Pharmacists can be also tasked with the detection of drug problems. One of the significant problems affecting older patients is polydrug use, i.e., taking at least five drugs per day (including preparations used in unconventional medicine) [34,35]. This phenomenon is intensified because in Poland, patients receive medicines from different physicians: specialists and a general practitioner. In the present study, 88.0% of pharmacists declare the detection of polypharmacy. However, there is a lack of public funding for drug review programs in community pharmacies. It is worth pointing out that the percentage of pharmacists who declared their willingness to provide services aimed at detecting polypharmacy, detecting drug interactions with dietary supplements, and detecting the prescribing cascade was the highest among the youngest pharmacists (under 35). This means that younger pharmacists are more open to pharmaceutical care and have fewer concerns about taking responsibility for the patient. It also seems that during their studies they were better prepared to provide this service.

A pharmacist can also help patients who take multiple prescription and over-the-counter (OTC) medications and can detect interactions between prescription medications and dietary supplements. This is important because the statistical data show that in Poland in each age group, the percentage of people using prescription drugs is similar to the European average (13.8% and 16.7% respectively), but the percentage of people using OTC far exceeds this average [36]. A high willingness to provide pharmaceutical care services related to pharmacotherapy monitoring among pharmacists in Poland indicates that community pharmacists can significantly contribute to patient care, improve patients’ compliance, and help physicians in more effective pharmacotherapy management. 

In this study, age and location of the pharmacy were the most important factors associated with pharmacists’ attitudes toward physician–pharmacist collaboration. Pharmacists under the age of 55 more often declared their willingness to provide six out of eight analyzed pharmaceutical care services, e.g., advice on lifestyle changes in chronic disease pharmacotherapy and compliance monitoring pharmacotherapy. This finding may result from the fact that younger pharmacists are more open to changes, and during their education they have obtained basic information in the field of interprofessional cooperation. In addition, younger generations are leaders of change, and younger pharmacists may be more willing to increase their role as pharmacists in the Polish healthcare system. Pharmacists working in rural areas were less likely to declare their willingness to educate patients in the use of medical equipment, pharmacotherapy, monitoring compliance with medical recommendations, and detecting polypharmacy. Those working in rural areas may have limited resources and are less likely to provide new types of services. Moreover, there is a limited number of physicians in rural areas [37].

Data presented in this study may be used by policymakers and scientific societies to develop guidelines on physician–pharmacist collaboration. Patient education and pharmacotherapy management should be considered priority pharmaceutical care services implemented in Poland. Moreover, this study pointed out that pharmacists aged below 55 years are more likely to provide pharmaceutical care services, and this group should be considered to be a leader of the change in the healthcare in Poland. In Poland, there are about 11,900 community pharmacies with over 25,900 pharmacists [25]. It is estimated that over 24% of all registered pharmacists in Poland are aged 60 years and over [25]. The retirement age in Poland is 60 years for women and 65 for men, so a significant proportion of pharmacists in Poland have reached or are close to reaching retirement age [25].

This study was limited to community pharmacists from one of the largest franchise chain pharmacy networks in Poland, so data from independent (self-governed) pharmacies are not included. Results cannot be generalized to all community pharmacists in Poland. A convenience sample size was used. Willingness to cooperate between pharmacists and physicians was based on self-declared responses, and medical records on the current state of physician–pharmacist collaboration were not analyzed. Nevertheless, this is one of the largest studies on physician–pharmacist collaboration in Poland.

## 5. Conclusions

Community pharmacists from one of the largest franchise chain pharmacy networks in Poland declared strong support for interprofessional collaboration and implementation of pharmaceutical care. Patient education, pharmacotherapy monitoring, and drug reviews were the most common pharmaceutical services that pharmacists would like to provide. Age and location of the pharmacy were the most important factors associated with pharmacists’ perceptions of physician–pharmacist collaboration. There is a need to develop evidence-based guidelines on physician–pharmacist collaboration that will meet the expectations of different groups of healthcare workers.

## Figures and Tables

**Table 1:** Pharmacists’ perceptions of the physician–pharmacist collaboration (n = 635).

Variable	Pharmacists n = 635	
	n	%
**There is a need for physician–pharmacist collaboration?**		
strongly agree	495	78.0
rather agree	128	20.2
rather disagree	4	0.6
strongly disagree	3	0.5
I do not know/Neither agree nor disagree	5	0.8
**The pharmacist can help the physician in patient care and the selection of optimal pharmacotherapy**		
strongly agree	424	66.8
rather agree	178	28.0
rather disagree	14	2.2
strongly disagree	1	0.2
I do not know/Neither agree nor disagree	14	2.2
**In your opinion, do physicians know the competencies of pharmacists resulting from the Polish law?**		
yes	35	5.5
no	508	80.0
I do not know/Difficult to tell	92	14.5
**What pharmaceutical care services can be provided by a pharmacist?**		
patient education on the use of medical equipment (e.g., glucometer, nebulizer)	571	89.9
counseling on lifestyle changes in chronic diseases	503	79.2
pharmacotherapy and adherence monitoring	394	62.0
pharmacotherapy and compliance monitoring	399	62.8
pharmaceutical counseling in minor health problems	351	55.3
detection of polypharmacy in patients receiving medication from different physicians	559	88.0
detection of interactions between drugs prescribed by a physician and dietary supplements self-ordered by the patient	544	85.7
detection of prescribing cascade and recommendations for limiting the number of drugs	356	56.1
**Which group of patients can benefit the most from physician–pharmacist collaboration?**		
patients visiting general practitioners	438	69.0
patients visiting specialized outpatient clinics	87	13.7
patients under hospital treatment	102	16.1
other groups of patients	8	1.3
**Areas requiring changes to facilitate physician–pharmacist collaboration**		
development of guidelines and recommendations on physician–pharmacist collaboration	597	94.0
inclusion of physician–pharmacist collaboration in medical education programs	520	81.9
providing public funding for physician–pharmacist collaboration	552	86.9

**Table 2:** Sociodemographic differences in the perception of the need for physician–pharmacist collaboration.

Variable	Conviction about the Need for Physician–Pharmacist Collaboration		Belief That the Pharmacist Can Help the Physician in Patient Care	
	n (%)	*p*	n (%)	*p*
**Gender**				
female	508 (98.8)	**0.01**	493 (95.9)	**0.01**
male	115 (95.0)		109 (90.1)	
**Age**				
<35	181 (98.9)	0.3	177 (96.7)	0.06
35–54	369 (98.1)		357 (94.9)	
55 and over	73 (96.1)		68 (89.5)	
**Having a pharmaceutical specialization**				
yes	200 (98.5)	0.6	190 (93.6)	0.4
no	423 (97.9)		412 (95.4)	
**Community pharmacies location**				
rural area	15 (93.8)	0.5	14 (87.5)	**0.01**
city <100,000 residents	317 (98.1)		306 (94.7)	
city from 100,000 to 50,000 residents	165 (98.8)		165 (98.8)	
city >500,000 residents	126 (97.7)		117 (90.7)	

Statistically significant values are bolded.

**Table 3:** Sociodemographic differences in the perception of pharmaceutical care services that can be performed by a pharmacist.

Variable	Patient Education on the Use of Medical Equipment (e.g., Glucometer, Nebulizer)		Counseling on Lifestyle Changes in Chronic Diseases		Pharmacotherapy and Adherence Monitoring		Pharmacotherapy and Compliance Monitoring	
	n (%)	*p*	n (%)	*p*	n (%)	*p*	n (%)	*p*
**Gender**								
female	467 (90.9)	0.1	411 (80.0)	0.3	309 (60.1)	**0.04**	323 (62.8)	0.9
male	104 (86.0)		92 (76.0)		85 (70.2)		76 (62.8)	
**Age**								
<35	160 (87.4)	0.2	143 (78.1)	**0.01**	118 (64.5)	**0.002**	115 (62.8)	**0.02**
35–54	345 (91.8)		309 (82.2)		243 (64.6)		247 (65.7)	
55 and over	66 (86.8)		51 (67.1)		33 (43.4)		37 (48.7)	
**Having a pharmaceutical specialization**								
yes	180 (98.7)	0.5	163 (80.3)	0.6	114 (56.2)	**0.04**	130 (64.0)	0.7
no	391 (90.5)		340 (78.7)		280 (64.8)		269 (62.3)	
**Community pharmacy location**								
rural area	11 (68.8)	**0.02**	10 (62.5)	0.4	11 (68.8)	0.06	7 (43.8)	**<0.001**
city <100,000 residents	290 (89.8)		257 (79.6)		192 (59.4)		183 (56.7)	
city from 100,000 to 50,000 residents	155 (92.8)		134 (80.2)		98 (58.7)		111 (66.5)	
city >500,000 residents	115 (89.1)		102 (79.1)		93 (72.1)		98 (76.0)	
**Variable**	**pharmaceutical counseling in minor health problems**		**detection of polypharmacy**		**detection of interactions between drugs and dietary supplements**		**detection of prescribing cascade and recommendations for limiting the number of drugs**	
	n (%)	*p*	n (%)	*p*	n (%)	*p*	n (%)	*p*
**Gender**								
female	282 (54.9)	0.7	462 (89.9)	**0.003**	447 (87.0)	0.06	283 (55.1)	0.3
male	69 (57.0)		97 (80.2)		97 (80.2)		73 (60.3)	
**Age**								
<35	113 (61.7)	0.07	167 (91.3)	**0.01**	167 (91.3)	**0.01**	118 (64.5)	**<0.001**
35–54	202 (53.7)		333 (88.6)		318 (84.6)		211 (56.1)	
55 and over	36 (47.4)		59 (77.6)		59 (77.6)		27 (35.5)	
**Having a pharmaceutical specialization**								
yes	103 (50.7)	0.1	173 (85.2)	0.1	174 (85.7)	0.9	108 (53.2)	0.3
no	248 (57.4)		386 (89.4)		370 (85.6)		248 (57.4)	
**Community pharmacy location**								
rural area	9 (56.3)	0.1	10 (62.5)	**0.01**	13 (81.3)	0.4	9 (56.3)	**0.04**
city <100,000 residents	164 (50.8)		282 (87.3)		270 (83.6)		176 (54.5)	
city from 100,000 to 50,000 residents	100 (59.9)		153 (91.6)		147 (88.0)		85 (50.9)	
city >500,000 residents	78 (60.5)		114 (88.4)		114 (88.4)		86 (66.7)	

Statistically significant values are bolded.

**Table 4:** Factors associated with the perception of the need for physician–pharmacist collaboration.

Variable	Conviction about the Need for Physician–Pharmacist Collaboration		Belief That the Pharmacist Can Help the Physician in Patient Care	
	OR (95% CI)	*p*	OR (95% CI)	*p*
**Gender**				
female	4.62 (1.49–15.33)	**0.01**	2.95 (1.35–6.48)	**0.01**
male	Reference		Reference	
**Age**				
<35	6.41 (0.85–48.22)	0.07	3.43 (1.00–11.77)	0.05
35–54	3.38 (0.72–15.92)	0.1	2.21 (0.85–5.74)	0.1
55 and over	Reference		Reference	
**Having a pharmaceutical specialization**				
yes	1.98 (0.44–8.83)	0.4	0.9 (0.39–2.06)	0.8
no	Reference		Reference	
**Community pharmacy location**				
rural area	0.58 (0.05–6.43)	0.7	0.91 (0.17–4.85)	0.9
city <100,000 residents	1.52 (0.36–6.32)	0.6	2.08 (0.94–4.56)	0.07
city from 100,000 to 50,000 residents	1.96 (0.32–12.17)	0.5	8.19 (1.78–37.66)	**0.01**
city >500,000 residents	Reference		Reference	

Statistically significant values are bolded.

**Table 5:** Factors associated with the perception of pharmaceutical care services that can be provided by a pharmacist.

Variable	Patient Education on the Use of Medical Equipment (e.g., Glucometer, Nebulizer)		Counseling on Lifestyle Changes in Chronic Diseases		Pharmacotherapy and Adherence Monitoring		Pharmacotherapy and Compliance Monitoring	
	OR (95% CI)	*p*	OR (95% CI)	*p*	OR (95% CI)	*p*	OR (95% CI)	*p*
**Gender**								
female	1.72 (0.93–3.19)	0.08	1.30 (0.80–2.10)	0.3	0.67 (0.43–1.04)	0.07	0.94 (0.61–1.43)	0.9
male	Reference		Reference		Reference		Reference	
**Age**								
<35	0.84 (0.34–2.05)	0.7	2.08 (1.07–4.05)	**0.03**	2.16 (1.19–3.94)	**0.01**	2.06 (1.12–3.77)	**0.02**
35–54	1.43 (0.64–3.23)	0.4	2.53 (1.40–4.55)	**0.002**	2.28 (1.34–3.88)	**0.002**	2.22 (1.30–3.80)	**0.004**
55 and over	Reference		Reference		Reference		Reference	
**Having a pharmaceutical specialization**								
yes	0.72 (0.39–1.32)	0.3	1.30 (0.81–2.09)	0.3	0.83 (0.57–1.22)	0.3	1.28 (0.86–1.89)	0.2
no	Reference				Reference		Reference	
**Community pharmacy location**								
rural area	Reference		Reference		0.88 (0.28–2.80)	0.8	Reference	
city <100,000 residents	3.77 (1.21–11.72)	**0.02**	2.02 (0.70–5.87)	0.2	0.53 (0.34–0.83)	**0.01**	1.50 (0.54–4.19)	0.4
city from 100,000 to 50,000 residents	5.20 (1.52–17.78)	**0.01**	2.01 (0.67–6.04)	0.2	0.50 (0.30–0.83)	**0.01**	2.25 (0.79–6.46)	0.1
city >500,000 residents	3.35 (1.03–11.59)	**0.04**	1.93 (0.63–5.90)	0.2	Reference		3.71 (1.26–10.94)	**0.02**
**Variable**	**pharmaceutical counseling in minor health problems**		**detection of polypharmacy**		**detection of interactions between drugs and dietary supplements**		**detection of prescribing cascade and recommendations for limiting the number of drugs**	
	OR (95% CI)	*p*	OR (95% CI)	*p*	OR (95% CI)	*p*	OR (95% CI)	*p*
**Gender**								
female	0.90 (0.60–1.36)	0.6	2.40 (1.37–4.20)	**0.002**	1.61 (0.95–2.75)	0.08	0.82 (0.54–1.25)	0.4
male	Reference		Reference		Reference		Reference	
**Age**								
<35	1.61 (0.89–2.90)	0.1	2.76 (1.20–6.36)	**0.02**	3.72 (1.64–8.45)	**0.002**	3.74 (2.02–6.90)	**<0.001**
35–54	1.20 (0.71–2.02)	0.5	2.12 (1.07–4.20)	**0.03**	1.85 (0.96–3.57)	0.07	2.54 (1.48–4.38)	**<0.001**
55 and over	Reference		Reference		Reference		Reference	
**Having a pharmaceutical specialization**								
yes	0.87 (0.61–1.26)	0.5	0.79 (0.45–1.39)	0.4	1.33 (0.78–2.27)	0.3	1.13 (0.78–1.65)	0.5
no	Reference		Reference		Reference		Reference	
**Community pharmacy location**								
rural area	Reference		Reference		Reference		Reference	
city <100,000 residents	0.82 (0.30–2.27)	0.7	4.08 (1.34–12.47)	**0.01**	1.07 (0.28–4.04)	0.9	0.85 (0.30–2.43)	0.8
city from 100,000 to 50,000 residents	1.16 (0.41–3.31)	0.8	5.65 (1.70–18.77)	**0.01**	1.44 (0.36–5.72)	0.6	0.71 (0.24–2.07)	0.5
city >500,000 residents	1.23 (0.43–3.55)	0.7	4.23 (1.28–13.99)	**0.02**	1.53 (0.38–6.23)	0.6	1.48 (0.50–4.40)	0.5

Statistically significant values are bolded.

## Data Availability

Data are available from the corresponding author on reasonable request.
